# Bilateral Bertolotti's Syndrome: A Case Report of an Uncommon Presentation of Chronic Low Back Pain in an Elder Patient

**DOI:** 10.7759/cureus.26569

**Published:** 2022-07-05

**Authors:** Zineb Barkhane, Soukaina Belaaroussi, Mehdi Foudail

**Affiliations:** 1 Faculté de Médecine et de Pharmacie, Université Hassan II de Casablanca, Casablanca, MAR

**Keywords:** mega-apophysis, lumbosacral spine, low back pain, congenital anomaly, bertolotti's syndrome

## Abstract

Lower back pain caused by anatomical lumbosacral transitional vertebra is known as Bertolotti's syndrome. We present the case of a 65-year-old male with persistent chronic lower back pain with radiological evidence of an anatomical lumbosacral pseudo-joint bilaterally. The patient underwent conservative treatment with lidocaine and steroids that helped to improve and manage his symptoms. Our patient is an elderly one, and it is quite uncommon for the first appearance of Bertolotti's syndrome. Therefore, Bertolotti's syndrome is a rare cause of lower back pain, and clinicians should consider it in the differential diagnosis.

## Introduction

Bertolotti's syndrome (BS), or lumbosacral transitional vertebra (LSTV), is a congenital defect marked by an enlargement of the caudal lumbar transverse process or subsidence at L5/S1 leading to pseudoarticulation or fusion of the transverse process of L5 with either the sacrum or ilium [1]. The pseudoarthrosis can cause significant low back pain (LBP) and movement restrictions. Mario Bertolotti first described the syndrome in 1917, and he correlated LBP reported clinically by the patients with an enlarged transverse process discovered radiographically [2]. At first, 4% to 8% of the population was estimated to be impacted by this anatomic variance, although a higher frequency of 30% is estimated [3]. Herein, we present a unique case of bilateral BS in an old male who complained of persistent chronic LBP.

## Case presentation

A 65-year-old male with a complaint of persistent LBP was admitted to the clinic with an otherwise unremarkable medical history. His family doctor made a diagnosis of a muscle sprain and treated him with a non-steroidal anti-inflammatory and a proton pump inhibitor for the last three years with minimal relief of his symptoms. He had pain radiating from the lower lumbar back to the buttocks, mainly on his left side. The patient described the pain as aching and throbbing. On first evaluation, the patient was conscious and coherent, the vital signs were normal; temperature: 36.9°C, blood pressure: 130/80 mmHg, heart rate: 78/minute, respiratory rate: 18/minute, oxygen saturation: 98% on room air. On physical assessment, no redness, swelling, or heat was detected in his lower back. However, there was a focal tenderness at L4-S1 and along the sacrum. The straight leg raised test was negative; also the reflexes, sensations, distal muscular strength, and pulses were intact. Pain limited his lumbar spine's ability to fully bend and rotate. However, all the other systems' physical examinations were normal. In addition, a blood test was done, and the results of inflammatory markers were within the normal range.

**Figure 1:**
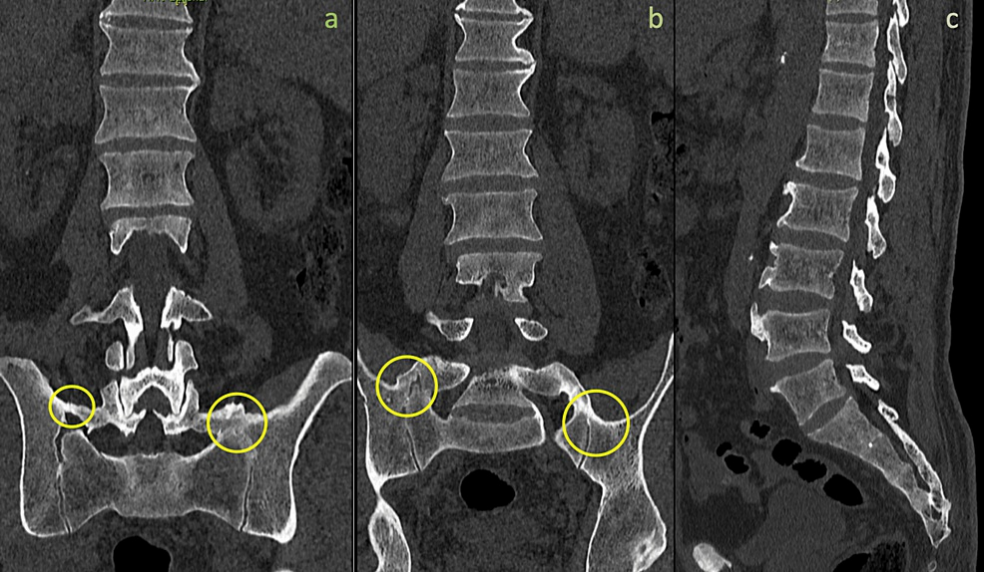
Coronal view (a and b) of lumbosacral spine CT revealing bilateral transverse mega-apophysis of L5 (yellow circles) articulating with the sacrum and ilium along with lumbar osteophytes. Sagittal view (c) detecting lumbar osteophytes CT: computed tomography

**Figure 2:**
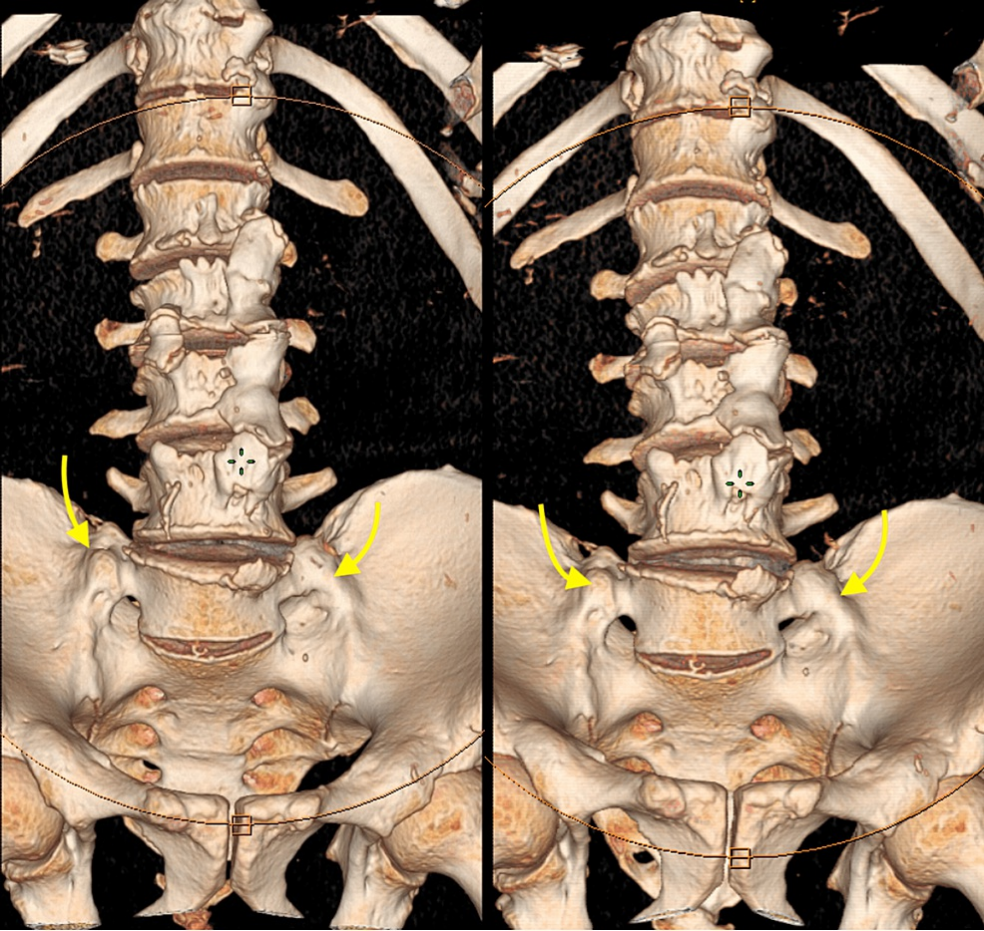
3D reconstruction of lumbosacral spine CT showing bilateral transverse mega-apophysis of L5 (yellow arrows) articulating with the sacrum and ilium. 3D: three-dimensional, CT: computed tomography

To determine the anatomic variants and the potential existence of fusion between the lumbosacral transitional vertebrae (LSTV) and the sacrum, also called pseudoarthrosis, a computed tomography (CT) scan (Figure 1) and a three-dimensional (3D) reconstruction (Figure 2) was conducted, which showed fusion between LSTV and sacrum bilaterally. Although the imaging findings were present bilaterally, the patient's left side displayed considerable discomfort. The differential diagnosis should always include facet and sacroiliac joint disease, but finally, our results from clinical exam and radiologic imaging pointed to BS.

Under fluoroscopic guidance, the patient was injected with 1 mL of lidocaine and 35 mg of triamcinolone in the pseudoarticulation between the transverse process and the sacrum. He immediately showed total pain relief afterward. During one month visit, he was provided physical therapy to help with mobilization, core strengthening, and stretching. At his three- and six-month follow-ups the patient did not report any symptoms.

## Discussion

BS is a developmental anomaly of the spine that usually develops when the first sacral segment and the last lumbar vertebra's lengthened transverse process combine with the sacrum and ilium [4]. It is first described in the early 20^th^ century [2], and Its prevalence in the general population has been estimated to be between 4% and 30%. 13% with an LSTV are generally asymptomatic, but only 4%-8% of patients are diagnosed, and 18.5% of them are below 30 years old [5]. In symptomatic cases of BS, the cause of pain is unknown. The etiology could be the fusion between the transverse process and the sacrum, as well as the associated degenerative alterations.

On the other hand, the fused transitional vertebra may cause instability above the fusion level. It is often challenging to accurately diagnose BS because of a lack of specific clinical signs. Typical findings in BS patients are a non-specific general back discomfort and a restriction in ranges of movement [6]. Some authors have stated that there is no association between the transitional vertebra and back pain [7,8], while others like Aihara et al. [9] discovered that the level just above the LSTV had significantly greater disk degeneration and thinner, weaker iliolumbar ligaments, which function to stabilize the lumbar spine in a torsional direction. The presence of LSTV likely causes hypermobility and altered load-bearing, which concentrate aberrant rotational and compressive stresses on the neighboring vertebra and increase the susceptibility to degenerative alterations [10,11]. It is believed that the onset of arthritic changes, disc herniation, disc degeneration, or spinal canal and foraminal stenosis led to the development of LBP [12]. Otani et al. [13] discovered that patients with LSTV experienced disc herniations more frequently (17% vs. 11%) and at a younger age (35 vs. 59) than those without LSTV. Furthermore, investigations have demonstrated that foraminal stenosis, disc degeneration, and facet degeneration are more common in patients with LSTV than in people without LSTV [14]. In 1984, Castellvi et al. [15] suggested a 30° angled anteroposterior view for a radiographic classification system identifying four types of LSTV based on morphologic characteristics (Table 1).

**Table 1:** Castellvi [15] classification of lumbosacral transitional vertebrae

Types	Morphology
I	Dysplastic transverse process (unilateral or bilateral)
II	Incomplete lumbarization/sacralization (unilateral or bilateral)
III	Complete lumbarization/sacralization (unilateral or bilateral)
IV	Combination (Types II and III)

According to the studies, LSTV type II-IV are linked to BS; although type I shows no symptoms, the majority of patients are discovered by coincidence. Type III is present in 8.3%-11.5% of the population [15,16]. LSTV anatomical variations are seen in varying degrees of frequency, and their incidence appears to differ by gender [15,16]. Nardo et al. [16], in particular, noted that males have a higher prevalence of LSTV than females.

A congenital lumbosacral vertebra with unilateral or bilateral enlargements of the transverse processes forming a mega-apophysis along with a fusion with the sacrum is the typical finding in X-rays imaging. The X-rays imaging can be used in conjunction with CT, which can provide a clearer vision of the implicated bone structures. In contrast, magnetic resonance imaging is helpful to make better images and evaluate a soft tissue around pseudoarticulation. There has been no definitive agreement by clinicians on managing patients with BS. The literature includes local injection, radiofrequency ablation, and surgery as treatment possibilities. A trial of conservative therapy with local steroid injection and oral analgesics is recommended in the first instance. Almeida et al. [17] observed that three out of five patients had only limited pain management after using radiofrequency ablation before surgical resection. Two patients saw a considerable improvement, and they were fully symptom-free [17]. Past literature has also reported surgery as a treatment often used for individuals who have failed medical treatments or who show symptoms of instability. This can be done unilaterally or bilaterally by removing the pseudoarticulation or by fusing the L4-S1 or L5-S1 joints [18]. In a short series of 16 individuals treated surgically, Santaveri et al. [19] observed that eight patients had transitional articulation excision and the other half had a posterolateral fusion. Interestingly, 10 patients experienced improved low back pain following surgery, and seven of the 10 had no back discomfort at all. The study's results recommend two surgical treatments for some Bertolotti's Syndrome patients [19]. Re-operations were necessary for three of the eight patients who underwent transitional articulation resection and three of the eight patients who underwent posterolateral fusion [19]. LSTV-related radiculopathy patients can benefit from nerve decompression surgery [20]. Translocating the nerve medially and removing the osteophyte or pseudoarticulation are both necessary for decompression [20]. As a result, surgical excision with decompression discloses some of the pathophysiologies of the cause of pain in BS.

Our case report clearly shows how BS can be easily missed. Young people are more likely to experience it unilaterally. However, our patient is old, and the presentation was bilateral. BS needs to be considered as one of the important causes of LBP in geriatric patients.

## Conclusions

Even though LSTV is commonly considered a developmental anomaly, the presentation of BS may appear in old patients, possibly due to anatomic exacerbation during the degenerative process. LBP is one of the most frequent complaints about seeking medical advice, and BS as a cause of LBP is not considered in diagnostic algorithms and remains an underdiagnosed entity. Physicians should include BS in the differential diagnosis for LBP in old patients.
